# The imperative for controlled mechanical stresses in unraveling cellular mechanisms of mechanotransduction

**DOI:** 10.1186/1475-925X-5-27

**Published:** 2006-05-03

**Authors:** Eric J Anderson, Thomas D Falls, Adam M Sorkin, Melissa L Knothe Tate

**Affiliations:** 1 Dept. of Mechanical & Aerospace Engineering, Case Western Reserve University, Cleveland, Ohio, USA; 2 Dept. of Biomedical Engineering, Case Western Reserve University, Cleveland, OH, USA

## Abstract

**Background:**

*In vitro *mechanotransduction studies are designed to elucidate cell behavior in response to a well-defined mechanical signal that is imparted to cultured cells, *e.g*. through fluid flow. Typically, flow rates are calculated based on a parallel plate flow assumption, to achieve a targeted cellular shear stress. This study evaluates the performance of specific flow/perfusion chambers in imparting the targeted stress at the cellular level.

**Methods:**

To evaluate how well actual flow chambers meet their target stresses (set for 1 and 10 dyn/cm^2 ^for this study) at a cellular level, computational models were developed to calculate flow velocity components and imparted shear stresses for a given pressure gradient. Computational predictions were validated with micro-particle image velocimetry (*μ*PIV) experiments.

**Results:**

Based on these computational and experimental studies, as few as 66% of cells seeded along the midplane of commonly implemented flow/perfusion chambers are subjected to stresses within ±10% of the target stress. In addition, flow velocities and shear stresses imparted through fluid drag vary as a function of location within each chamber. Hence, not only a limited number of cells are exposed to target stress levels within each chamber, but also neighboring cells may experience different flow regimes. Finally, flow regimes are highly dependent on flow chamber geometry, resulting in significant variation in magnitudes and spatial distributions of stress between chambers.

**Conclusion:**

The results of this study challenge the basic premise of *in vitro *mechanotransduction studies, *i.e*. that a *controlled *flow regime is applied to impart a *defined *mechanical stimulus to cells. These results also underscore the fact that data from studies in which different chambers are utilized can not be compared, even if the target stress regimes are comparable.

## Background

Mammalian cells inhabit a variety of biochemical and biophysical environments within the body, many of which are defined by exposure to distinct and dynamic fluid media. Flow of fluid plays a key role in mechanotransduction via direct transfer of mechanical forces from the fluid to the membranes of cells as varied as those found in the vascular endothelium [1], bone interstitium [2], and renal proximal tubules [3]. Whether induced by contraction of cardiac muscle, mechanical loading, or accumulation of renal filtrate, these flows create shear stresses at the fluid/cell interface that have been hypothesized to strain the cytoskeleton, trigger cellular force receptors and/or affect the conformation of membrane bound proteins implicated in numerous healthy, inflammatory, or disease state signaling pathways [4-6] Indirectly, local flows influence mechanochemical transduction by regulating the chemical environment that governs cell activity through both the early development of and subsequent remodeling of tissues [7-11]. By modulating chemokinetic gradients and osmotic pressure, fluid flow may affect receptor binding kinetics [12], membrane porosity [13] as well as chemotaxis [14,15]. The fluid-structure interactions at the cellular level of many tissues are poorly understood yet they appear to be universal across tissue types and may hold the key to unraveling mechanisms of mechanotransduction at a cellular and subcellular level. Knowledge of such mechanisms could be applied not only to understand etiology of different diseases but also to develop prophylactic measures to prevent such diseases [16-18].

Due to practical difficulties in studying fluid flow *in situ *during normal physiologic activity, cell perfusion chambers have been developed to simulate such physiologic fluid flow and to observe cellular responses *in vitro*. In particular, the pressure driven parallel-plate perfusion chamber design has been implemented [19-23] and optimized [24-27]. for application of known fluid shear stresses and correlation to cell activity and adaptation [28]. Variations of the parallel-plate chamber design have become commonplace in cell biological research and provide a basis for current *in vitro *modeling of physiologic flow regimes including those relevant to bone [20,21,28-31], articular cartilage [32], connective tissue [33], vascular endothelium [34], leukocyte recruitment [14,35], as well as pathologies specific to renal dysfunction [36], and respiratory distress [37]. In addition, flow perfusion chambers have been implemented to characterize cell-biomaterial interactions [27,38,39], improve tissue engineered implants [40], and develop novel biomedical applications [41]. While this approach has obvious advantages for investigating effects of fluid shear in diverse biomedical arenas, it is not known how well these *in vitro *flow chambers perform, *e.g*. in achieving a desired stress at the cell level or in emulating physiologic flow regimes [42].

The ability to study cells in a controlled environment which mimics the conditions found *in vivo *is essential to understanding many basic cellular mechanisms, such as the cellular response to applied shear stress. As computational models have been developed to predict flow within the examined chambers, it is also necessary to examine the flow experimentally. In order to use a parallel plate flow chamber as a test bed for further studies, it is essential to know how close the stresses actually imparted at the cellular level match the target stresses.

Hence, the purpose of this study was to compare flow regimes in three commercially available cell flow/perfusion chambers to evaluate their efficacy in providing a defined flow regime and shear stress to cultured cells. For each chamber, the principal velocity component and local shear stress imparted through fluid flow were calculated for a target shear stress of 1 and 10 dyn/cm^2 ^used typically for osteoblast stimulation [31] Special attention was paid to local flow regimes in the vicinity of cells within the chambers. Computational results for velocity were validated using microparticle image velocimetry (*μ*PIV) for cases with and without cells seeded in the chambers.

## Methods

### 3-D modeling

**Figure 1:**
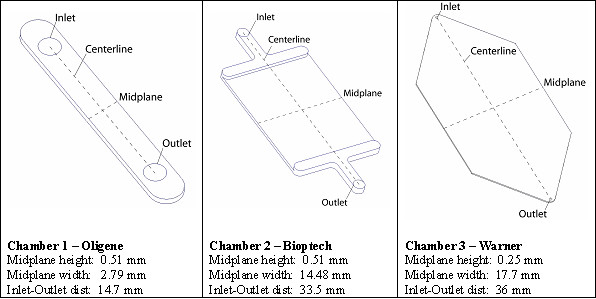
Schematic diagrams demonstrating characteristic dimensions of the flow chambers studied (not to scale).

Computational fluid models were created for three commercial cell flow/perfusion chambers (FCS, Oligene GmbH; FCS2, Bioptechs; RC-30 HV, Warner Instruments) to elucidate the effect of their specific design parameters on flow fields and resulting stress regimes that are imparted to cells seeded within the chambers (Figure 1). First, dimensions of all surfaces that define the fluid geometry (inlets, outlets, and chamber walls) were measured using a precision caliper and micrometer. Then the fluid was mapped to track the flow from inlet to outlet. Thereafter, for each commercial chamber, flow regimes were analyzed and compared for two target fluid shear stress magnitudes representative of those typically imparted to an osteoblastic monolayer (1, 10 dyn/cm^2^).

### Fluid meshing

The creation of the fluid mesh is critical to the computational analysis, as it delineates interfaces between fluid cavities as well as node locations where each calculation is made by the solver. Care was taken to place node locations in the critical areas throughout the model, in particular at flow transition areas such as at inlets, outlets, and points of flow expansion or contraction. This procedure not only ensures the accurate description of flow through the channels, but it also reduces the computational requirements of the simulation. Hence, the mesh includes only fluid volumes within the chamber itself and not flow volumes within inlet/outlet tubing or volumes outside of the device. Two sets of models were created, accounting for (*i*) the chamber geometries without cells seeded on the bottom surface (for all chambers), and (*ii*) the chamber geometries with an array of cells modeled on the lower surface of the flow chamber. Similar to previous studies on flow over cell-shaped protrusions, the cells were modeled as rigid spheroid protrusions on the chamber surface, with dimensions typical of osteocytes (height = 10 *μ*m, radius = 15 *μ*m) [43,45,50]. The number of nodes used in each chamber was 64000, 480000, and 89000 for chambers 1, 2, 3 respectively, where the average finite volume modeled was on the order of 10^-13 ^m^3^. Finally, the mesh, which provides a visual map of the flow geometry, was imported into a computational fluid dynamics (CFD) package (CFD-ACE, CFDRC), to allow for the definition of boundary conditions and simulation of flow regimes for targeted stress magnitudes.

### Computational fluid dynamics

For each chamber, the velocity profile and pressure variation were determined at the inlet and outlet, for a corresponding maximum target shear stress 1 and 10 dyn/cm^2^, at the location where cells are placed within the chamber (*i.e*. bottom surface of chamber). These were then applied as boundary conditions, to focus simulations on the interior of the chamber cavity where the cells are cultured. Using a discretization convective-upwind scheme, velocity profiles were calculated from the continuity equation and Navier-Stokes equations in three dimensions (3D),

∇·*u *= 0     (1)

*ρ *(*u*·∇*u*) = -∇*p *+ *μ*∇^2^*u *    (2)

where *u *is velocity vector, *μ *is the fluid viscosity, *p *is pressure, and *ρ *is density. Pressure and velocity at the center of each finite volume are decoupled by linear interpolation, where instabilities are avoided by averaging the Navier-Stokes equations for each volume face and relating the face velocity to the pressure gradient. Reynolds number, Re,



was also calculated for each case to further characterize the flow. Values calculated based on the mean velocity, *u*_*m*_, and hydraulic diameter, *D*_*h*_, at the midplane of the chambers are estimated to be on the order of 1 – 4. As this Re number falls well within the laminar region (laminar flow, Re<1400), viscous-dominated flows are anticipated. Using velocity components and pressure from above, the fluid shear stress, *τ*, at the surface of the chamber was calculated from the viscosity and rate of strain, ,

*τ*_*wall *_= *μ*     (4)

The perfusion medium was idealized as water with appropriate constant fluid properties: *μ *= 0.001 kg/m-s and *ρ *= 1000 kg/m^3^. A no-slip boundary condition was used for all chamber walls, and the inlet/outlet conditions were determined for standard pipe (tubing) flow with a laminar parabolic velocity profile and corresponding pressure gradient. Simulations were carried out using a finite-volume numerical method under steady flow conditions, with a convergence criterion of 0.0001, for the solution of each velocity component and pressure gradient per finite volume. The resulting calculations included 3D spatial resolution of the velocity profiles, pressure gradients along the flow direction (axial), and the shear stress at the bottom surface. These data were recorded for each chamber.

Node densities were increased at the center of the chamber in order to track flow and stress fields at higher resolution in the area where cells are seeded in mechanotransduction studies. The volume of fluid directly above this center section was isolated for each case and the velocity profile and pressure gradient were magnified in this section to increase resolution and to extract maximum and minimum values. Shear stresses experienced at the surface were then determined for each flow chamber. Thus, accurate comparisons could be made between global flow regimes in the commercial perfusion chambers as well as local flow regimes that impart stresses to cells within the chamber.

### *μ*-PIV validation

In order to validate the velocity and shear stress components found in the computational models, microparticle image velocimetry (*μ*PIV) techniques were performed to measure the rate of flow found within each chamber design. A Leica DMIRE-2 (Leica Microsystems, Inc, Bannockburn, IL) inverted epifluorescent microscope with integrated (hardware and software) Scan IM 100 × 120 automated stage (Marzhauser GmbH & CO, Wetzlar-Steindorf, Germany) and Retiga EXi camera (Q-Imaging, Burnaby, BC, Canada) were used to image TetraSpeck fluorescent microparticles (4 *μ*m diameter; excitation wavelengths 365/505/560/660 nm; emission wavelengths 430/515/580/680 nm; T-7283, Molecular Probes, Eugene, OR) as they traveled through the chamber in a 2.8 × 10^4 ^microspheres per ml DH_2_O suspension. As the suspension moved through the flow channel, an automated imaging routine (implemented in OpenLab 4.0.3, Improvision Inc, Lexington, MA) captured images of a grid containing the entire flow field. This procedure was then repeated 5 times consecutively to capture the maximum number of particles, and to minimize sampling error. This process was then repeated at several planes spaced 50–100 *μ*m apart through the depth the flow channel. The microspheres appeared in the images as streaked lines of varying length, where the length of the streak was equal to the distance traveled during the exposure time interval.

The same set of procedures was also used to perform another *μ*PIV study to determine any effects that seeded cells might have on flow fields within the chamber. The Oligene chamber (chamber 1) was implemented for this set of experiments. Degreased silica glass coverslips were etched with sodium hydroxide for 1 hour, and then covered with a 0.15 mg/mL solution of a collagen/acetic acid solution for 1 hour. After rinsing, MLO-Y4 osteocyte-like cells (a generous gift from Lynda Bonewald, University of Missouri-Kansas City) were seeded onto the coverslips at a density of approximately 5500 cells/cm^2^. The cells were then incubated for 48 hours before being fixed in a 3.7% solution of formaldehyde for 10 minutes.

The particle velocities within each chamber were calculated using a combination of image processing and symbolic mathematical manipulation software. After conversion to gray-scale, the images were auto-leveled in Adobe Photoshop CS (Adobe Systems, Inc.) to enhance contrast between the particle streaks and background noise. Image thresholding and particle analysis was completed using ImageJ 1.34 (NIH, Bethesda, MD). After exclusion parameters were applied to remove any artifacts (too large or small to be particles), the output data file for each image was processed using a Mathematica (Wolfram Research, Inc.) notebook file. Velocity was calculated as the distance traveled per duration of imaging (i.e. shutter speed). The sequential data at each point was combined to create an array of sample-averaged velocities, and used to generate a vector field depicting particle velocity through the entire flow field. The measured profiles were then compared to the calculated velocity components obtained from the CFD models for flow rates equivalent to the target shear stresses in order to validate the computational results.

Particular care was taken to ensure repeatability of trials as well as to minimize random error. Using the automated stage and OpenLab, the exact position of the flow channel (with respect to the stage adapter) was recorded in the software for each chamber. This allowed for the automations to be repeated using the same image coordinates each time. Any random errors that were introduced when capturing the particle streaks were minimized by running the automation five times consecutively for each focal depth, in order to capture the maximum number of particle streaks possible. Two-times binning, which acquires 2 × 2 adjacent pixels as one large pixel, was used to increase the speed of image (and particle streak) capture. Pixel size is 0.5 × 0.5 *μ*m for the 20× objective and 1.0 × 1.0 *μ*m for the 10× objective. Hence, binning, which is implemented to minimize any lag time in real-time imaging, could potentially introduce an error of 1–2% in measurement of microsphere displacement, *e.g*. considering a 100 *μ*m total displacement. During image processing, particle streaks attributable to background noise or particles not moving with the rest of the fluid flow were identified as being outside of the range of lengths possible for the given flow regime and were removed.

## Results

**Figure 2:**
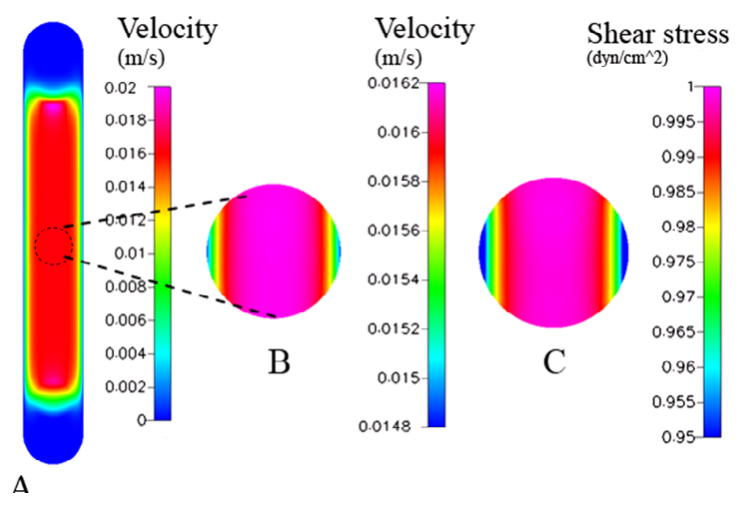
Chamber 1 (Oligene) – computational model predictions are shown for the (A) velocity profile [m/s] at the center of the chamber (maximum velocity), (B) velocity within the region of interest, and (C) wall shear stress [dyn/cm^2^] within the region of interest for cell mechanotransduciton studies.

**Figure 3:**
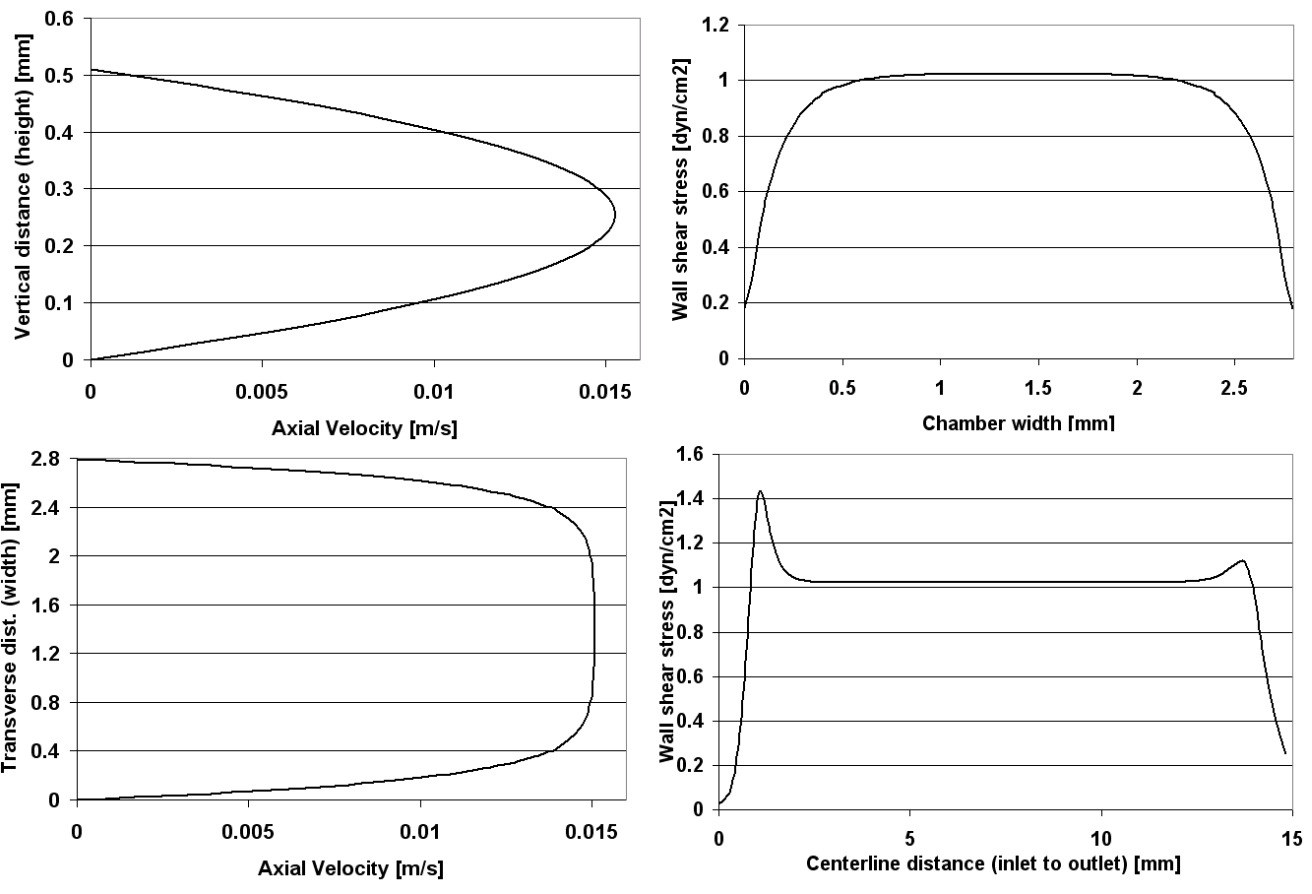
Chamber 1 (Oligene) – computational model predictions showing velocity profiles at midplane and wall shear stress profiles at the midplane and centerline of the chamber.

**Figure 4:**
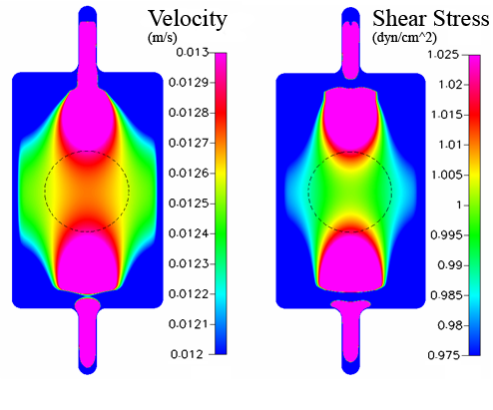
Chamber 2 (Bioptechs) – computational model predictions are shown for the velocity profile [m/s] at the center of the chamber (maximum velocity) and wall shear stress [dyn/cm^2^] along chamber surface, within the region of interest for cell mechanotransduciton studies.

**Figure 5:**
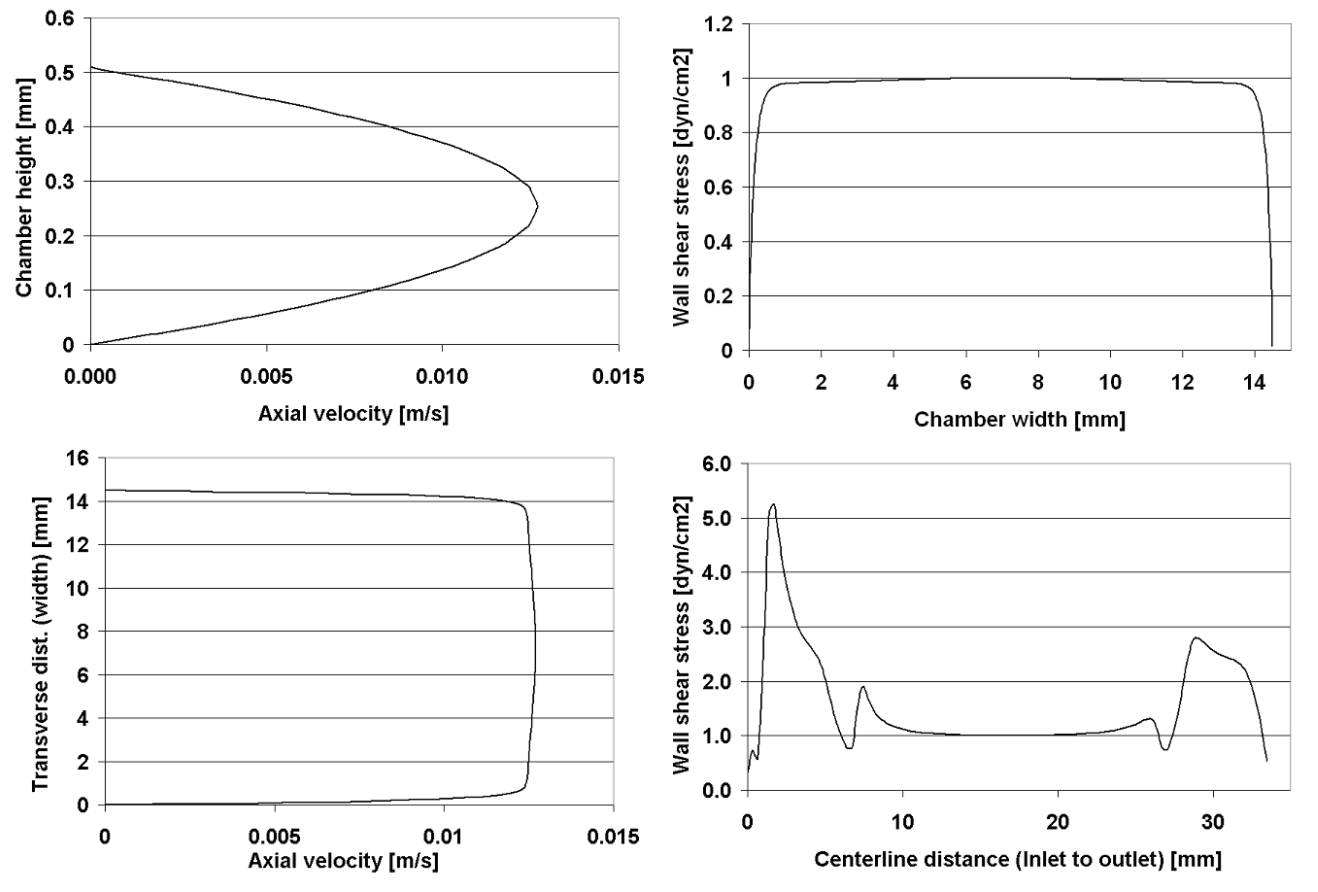
Chamber 2 (Bioptechs) – computational model predictions showing velocity profiles at midplane and wall shear stress profiles at the centerline of the chamber.

**Figure 6:**
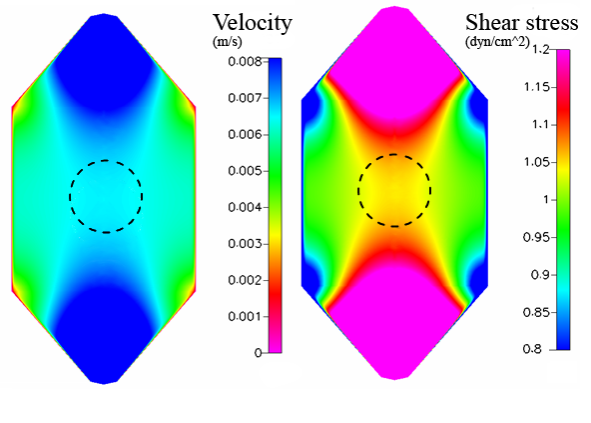
Chamber 3 (Warner) – computational model predictions are shown for the velocity profile [m/s] at the center of the chamber (maximum velocity) and wall shear stress [dyn/cm^2^] along chamber surface, within the region of interest for cell mechanotransduciton studies.

**Figure 7:**
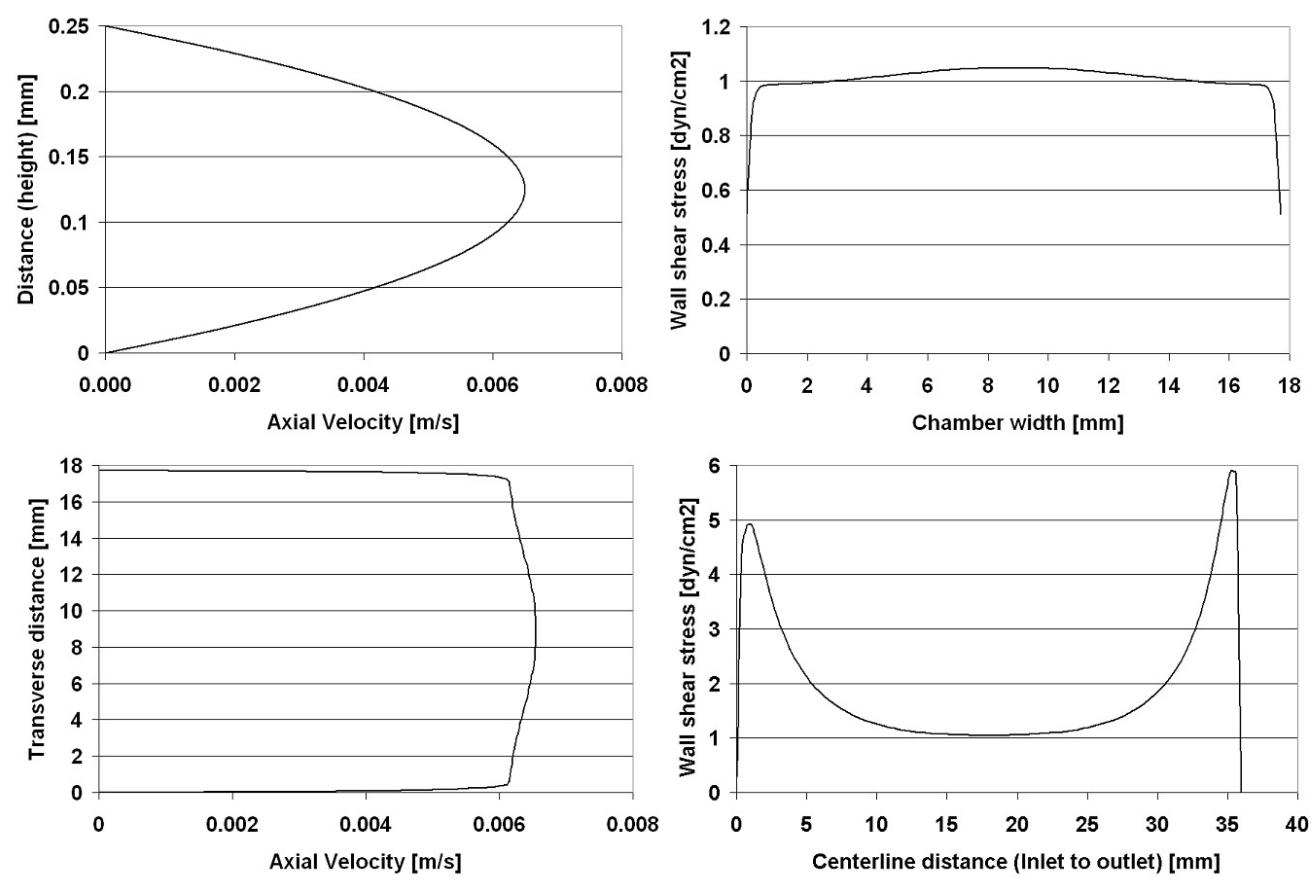
Chamber 3 (Warner) – computational model predictions showing velocity profiles at midplane and wall shear stress profiles at the centerline of the chamber.

**Figure 8:**
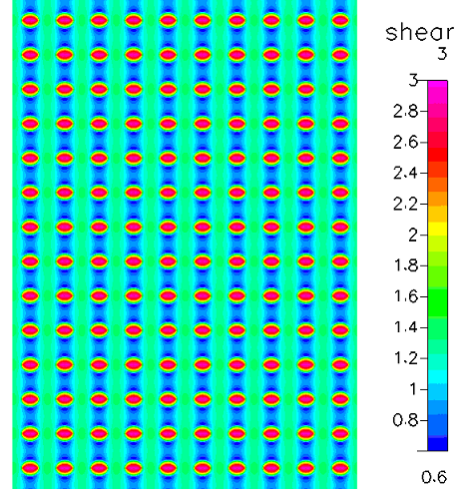
Case study based on computation model implementing geometry of Chamber 1 (Oligene) and including the cell monolayer on bottom surface. Looking from above, the shear stress [dyn/cm^2^] is mapped in region of interest for cell mechanotransduction studies.

**Figure 9:**
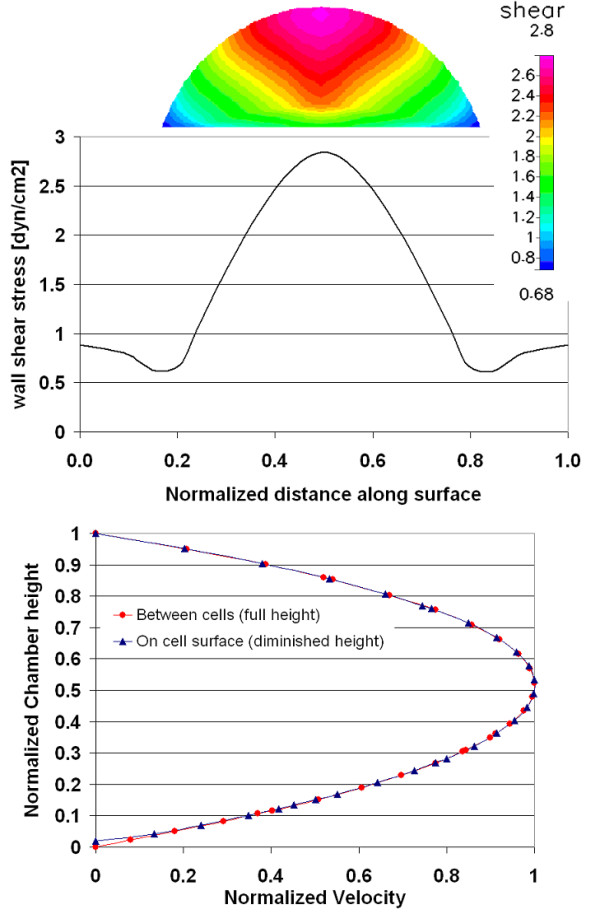
Experimental measurement of velocity profiles and corresponding shear stresses for the case study implementing the geometry of Chamber 1 (Oligene). The shear stress profile is depicted along the surface of the cells and velocity profiles are shown (a) over a cell surface (shortened chamber height) and (b) between cell array (normalized height), in region of interest where a cell monolayer is cultivated within the chamber.

In each chamber, the *velocity component *of the flow field varies as a function of location within the area of cell seeding. As a result, the local shear stress imparted to the cells varies as a function of location as well. Only a limited area is exposed to the target stress level (Table 2). Furthermore, the *range *of imparted wall shear stresses vary from chamber to chamber by up to 2-fold along the midplane without cells (Figures 2, 3, 4, 5, 6, 7). When cells are included within a specific chamber, the imparted stress at the wall (on cell body) increases by 3-fold (Figures 8, 9). Finally, the *location *of the area where the targeted shear stress (1 and 10 dyn/cm^2^) is achieved varies from chamber to chamber. Details for each chamber with a target stress of 1 dyn/cm^2 ^are described below; results with a greater target stress (10 dyn/cm^2^) yielded similar profiles, with increased magnitude, and are summarized at the end of this section.

**Table 1:** Flow rates needed for 1 dyn/cm^2 ^shear stress, magnification used and camera shutter speed used for each of the three chambers tested (including the Oligene chamber with the cells seeded on the coverslip). Note: The eyepiece objective of the microscope used was 1×.

**Chamber**	**Flow rate (ml/min) for 1 dyn/cm^**2**^**	**Magnification**	**Camera Shutter Speed (ms)**
**Oligene FCS**	0.774	20×	20
**Oligene FCS (with cells)**	0.774	20×	20
**Bioptechs FCS2**	3.624	10×	20
**Warner RC-30 HV**	1.278	10×	10

**Table 2:** Computational results: percentage of the region of interest for each chamber that is within the specified ranges of the target wall shear stress; definitions of midplane and centerline for each chamber are shown in Figure 1.

**Chamber**	**Region of interest**	**Within 5% of target stress**	**Within 10% of target stress**	**Within 50% of target stress**
**Oligene FCS**	Midplane	49%	72%	96%
	Centerline	75%	81%	92%
**Bioptechs FCS2**	Midplane	92%	94%	98%
	Centerline	35%	42%	67%
**Warner RC-30HVS**	Midplane	96%	97%	100%
	Centerline	10%	28%	58%

In the first chamber studied, the calculated velocity magnitudes remain constant along the centerline, with a maximum velocity of 0.0152 m/s for a Reynolds number of 4 (Figure 2). The corresponding wall shear stress magnitudes vary midplane, along the width of the chamber floor (which is narrower than the others studied, *i.e*. 2.8 mm as compared to ~14.5 and ~17.7 mm, for chambers 2 and 3 respectively). Looking into the depth of the chamber (Figure 3), wall shear stress magnitudes along the lower surface range from 0.2 – 1.05 dyn/cm^2 ^with a mean stress of 0.89 dyn/cm^2 ^(measured midplane between the inlet and outlet). Only 49% of midplane data points (evenly spaced) were within ±5% of the target shear stress; however 72% of the midplane data points fell within 10% of the target stress (Table 2). Shear stress peaks near the inlet/outlet of the chamber but remains nearly constant (1.02 dyn/cm^2^) along the centerline of the chamber. The resultant stress deviates increasingly from the target shear stress, with increasing distance from the centerline of the chamber.

**Figure 10:**
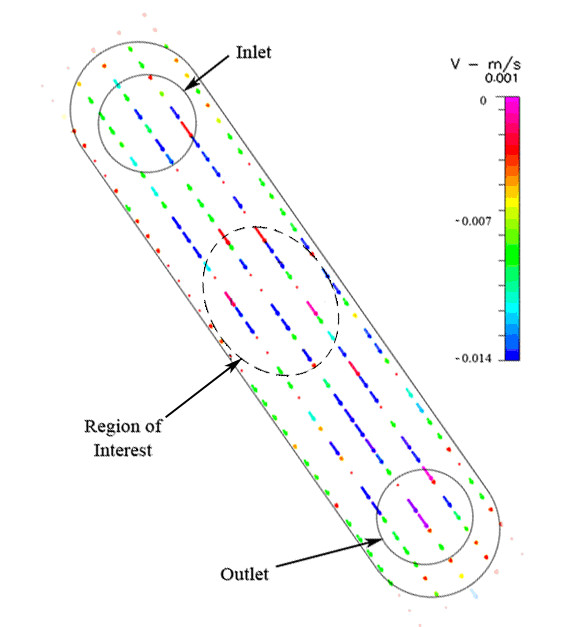
Experimental transverse velocity profile, of one focal plane, for the Oligene FCS chamber.

The experimentally measured flow profile shows a peak in fluid velocity at the center of the channel, which increases with proximity to the side walls (Figure 10). Furthermore, actual fluid velocities measured with PIV rarely reached target velocities (calculated as 0.0151 m/s and predicted to produce the 1 dyn/cm^2 ^target shear stress) in the area where cells are seeded (Table 3). None of the 26 data points examined was within ±10% or ±25% of the target velocity, and only 7 out of 26 (26.92%) data points were within ±50% of the target velocity (Table 3). It should be noted that the region of interest in this chamber is approximately one fourth of the size of the other two chambers (Oligene: 2 mm, Bioptechs and Warner: 8 mm). A similar number of data points were obtained by imaging the PIV experiments at a magnification of 20×.

**Table 3:** Experimental: number of data points within +/- 10, 50 and 100 % of the target value for each of the three chambers tested. The regions of interest used are depicted in Figures 10-12.

**Chamber**	**Within ±10%**	**Within ±25%**	**Within ±50%**
**Oligene FCS**	0%	0%	26.92%
# data points/total	(0/26)	(0/26)	(7/26)
**Bioptechs FCS2**	20.00%	73.33%	96.67%
# data points/total	(6/30)	(22/30)	(29/30)
**Warner RC-30HVS**	21.43%	39.29%	85.71%
# data points/total	(6/28)	(11/28)	(24/28)

### Chamber 2 (Bioptechs) – 1 dyn/cm^2^

In the second chamber studied flow profiles are dominated by the inlet and outlet expansion and nozzle zones, respectively (Figure 4). Here, the midplane velocity varies only slightly across the width of the chamber (14.4 mm), where the maximum velocity is 0.0127 m/s; however greater variations in velocity are found between the inlet and outlet (along centerline), corresponding to Reynolds number of 4 (Figure 5). The range in wall shear stress that would be experienced by the cells at the surface, along the midplane (0.15 – 1 dyn/cm^2^), is lower than that of the previous chamber at comparable locations. Ninety-four percent of the midplane region experiences stresses within ±10% of the target stress (Table 2); the mean stress comprises 0.91 dyn/cm^2^. However, along the centerline, only 35% of the region is within ±10% of the target, and only 67% within ±50% of the target, respectively.

**Figure 11:**
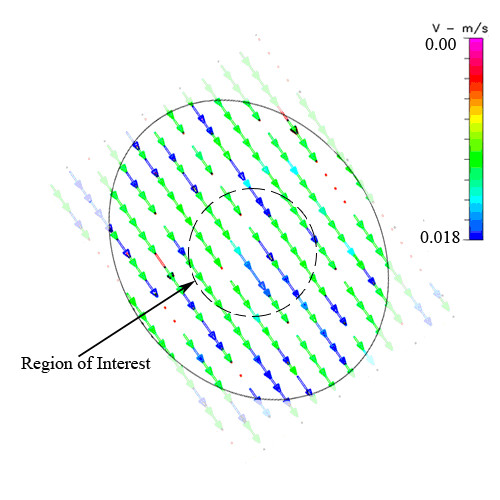
Experimental transverse velocity profile, of one focal plane, for the Bioptechs FCS2 chamber. Note: for this chamber, the area viewable for imaging differs from the gasket geometry; hence, the outer boundary indicates the area that is observable under the microscope.

Experimentally measured flow velocities are relatively uniform in this chamber (Figure 11); this is due in part to the fact that the region of interest is small in comparison to the length of the entire channel (only 30 out of 183 data points), which aids in maintaining uniform flow velocity magnitudes. For this chamber, a 0.0118 m/s target velocity is necessary to achieve 1 dyn/cm^2 ^of shear stress. While only 6 out of 30 (20%) examined data points were within +/- 10% of the target velocity, 22 out of 24 (73.33%) were within +/- 25% of the target velocity. Nearly all of the data points, 29 out of 30 (96.67%) were within +/- 50% of the target velocity (see Table 3).

### Chamber 3 (Warner) – 1 dyn/cm^2^

The design of the third chamber represents a geometric compromise between the first two chambers studied. The flow profile across the midplane of this chamber (Figure 6) is similar to that of the second chamber; predicted flow velocities are relatively uniform across the width of the chamber, reaching a maximum of 0.00637 m/s (Reynolds number of 1). Similar to the previous chambers, there is little variance in predicted wall shear stress on the midplane. However, strong variation is predicted along the centerline from inlet to outlet. In this case, the shear stress across the bottom surface at the midplane varies from 0.5 – 1.05 dyn/cm^2^, with a mean stress of 1.01 dyn/cm^2 ^(Figure 7). While 97% of the midplane region is within ±10% of the target stress, only 10% of the region along the centerline exhibits a shear stress within ±5% of the target, and only 58% of the latter region shows a shear stress within ±50% of the intended shear stress (Table 2).

**Figure 12:**
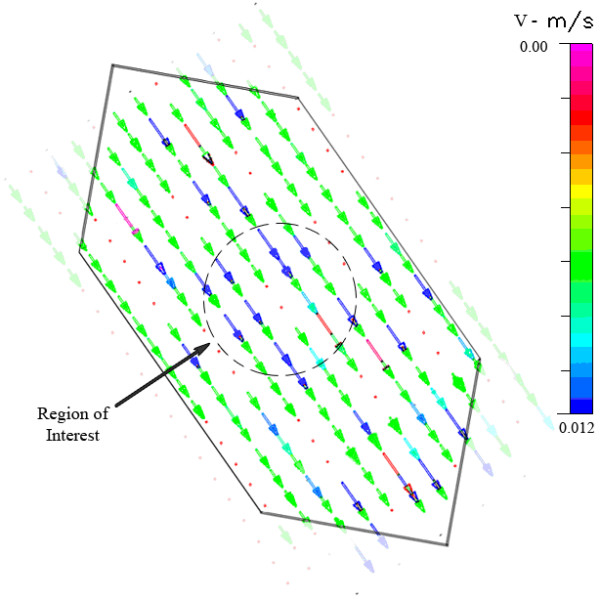
Experimental transverse velocity profile, of one focal plane, for the Warner RC-30 HV chamber.

At a characteristic height, the flow profile in this chamber (Figure 12) follows the flow pattern of the idealized parallel plate chamber. The fluid velocity is at its maximum through the central portion of the flow channel and tapers off as it approaches the side walls. In this chamber, the area of interest is approximately the same size as that in the Bioptechs chamber, resulting in a similar number of data points (28) being examined. The target velocity required to achieve 1 dyn/cm^2 ^shear stress is approximately 0.0065 m/s. Only 6 out of 28 points (21.43%) were measured within +/- 10% of the target velocity, and just 11 out of 28 points (39.29%) were within +/- 25% of the target velocity. However, 24 out of 28 points (85.71%) were within +/- 50% of the target velocity (Table 3).

### Results with cell monolayer – 1 dyn/cm^2^

To address any potential effects of cells on prevailing flow regimes, computational predictions are also reported for a case study including cells that are seeded "virtually", on the lower surface of the chamber. Data from case studies using the Oligene chamber geometry is reported and is representative of the results from all three chambers. Inclusion of the cell monolayer in the computational analysis resulted in an almost three-fold increase in applied shear stress on the cell surface (2.8 dyn/cm^2^, Figure 8); the mean stress was almost twice as high as the target stress. It should be noted that the chamber height decreases due to the presence of the cell layer; this results in an increase in velocity with respect to height of the flow volume and results in an increase in imparted stress (Fig 9).

In addition, experimental data was obtained for the Oligene FCS chamber with MLO-Y4 cells seeded on the coverslip. As the particles used for imaging fluid displacements are on the same order of magnitude as cell dimensions, some interactions may have occurred between the beads and cell layer, particularly in the direction perpendicular to flow. The results obtained at a depth of midchannel and upwards correspond well to the computationally predicted cross sectional profile. The velocity is at a maximum close to the center height, decreasing in a near parabolic manner towards the top surface of the channel.

### Results with target stress of 10 dyn/cm^2^

In a final stage of the analysis, higher target shear stress regimes, *i.e*. 10 dyn/cm^2^, were applied to evaluate efficacy of the three chambers in achieving target shear stresses typical for osteoblast and endothelial cell research [31,34,44] Increasing the target shear stress results in similar flow patterns across the chamber, but the range of observed shear stress magnitudes increases. For all chambers, the velocity profile essentially scales with the increase in target stress (from 1 to 10 dyn/cm^2^). Thus, profiles depicted in the figures are appropriate for understanding chamber performance for various flow rates. In each case, these results show an increase from those calculated for the lower target stress levels in the first stage of the study, and hence, a pattern emerges where the range of shear stress experienced may be proportional to the increase in target stress or flow range.

## Discussion

Flow/perfusion chambers are designed to impart a constant target shear stress to cells seeded within. In this study, computational fluid dynamics models and experimental fluid dynamics studies were implemented to predict and to measure shear stresses achieved at the cell level when implementing three commercially available cell flow/perfusion chambers. Both computational predictions, as well as *μ*piv measurements of actual fluid displacements, showed that the fluid velocity magnitude defining the local mechanical environment of cells is poorly controlled and is location dependent. This results in flow-imparted cellular shear stresses that are highly variable and rarely reach the magnitudes of 1 and 10 dyn/cm^2 ^set as targets in the context of this study. Furthermore, the mechanical stimulus imparted to cells within a chamber is location dependent, so that two neighboring cells may be exposed to significantly different stresses. In fact along the midplane, between 49% and 96% (depending on the chamber) of imparted stresses are within ±5% of the target magnitude when cells are not included. However, stresses on the cell surface itself can reach as high as 280% of the target value if the cell monolayer is included explicitly in the model. This challenges the basic assumption that all cells in a monolayer experience the desired target stress set using the idealized formula for calculating shear stress at the wall in a parallel plate model, which is the basic paradigm underlying most parallel plate flow chamber designs and their implementation. These trends, *i.e*. the failure to achieve target stresses and the spatial dependence of stress magnitudes across the cell monolayer, were observed in all three flow/perfusion chambers tested. In contrast to these common trends, there was little commonality in flow regimes or shear stresses magnitudes imparted to the cells between the chamber devices studied. Based on these data, not only is calibration of each flow chamber imperative to "tune" or to maximize the possibility of achieving the target stress over the area on which cells are seeded, but also, prior to comparing data between systems, one must take into account intrinsic differences in the flow regimes produced by the different devices.

Cell perfusion chambers have been developed to simulate physiologic fluid flow *in vitro *and to study the role of fluid flow in mechanotransduction [19,20,22,23,46]. However, this study evaluates how well these systems perform with respect to achieving the target stress to be imparted to the cell. Among the tested commercial perfusion chambers, all three of the flow regimes showed considerable differences in the flow patterns and shear stresses achieved. The magnitudes of velocity in each chamber varied according to the critical dimensions, as expected, however the shear stresses imparted to the specimen in each chamber fell within dissimilar ranges. The target shear stress was achieved only within a small area of each commercial chamber and the location of this area varied from chamber to chamber. Thus, comparisons of outcome measures for a specific cell response may not be appropriate if different chambers were used to impart stresses to the cell. In addition, the target stress value may not always be located in the same area in different chambers; therefore, the observation of a specific point for comparison would prove inaccurate since various flow chambers could cause a variety of desired shear stress locations.

The presence and location of seeded cells, as well as the point of observation, are critical. Interestingly, computational model predictions of imparted shear stresses increase by almost three-fold when cells are accounted for in flow geometry delineation; this corroborates data reported in the literature [45]. Furthermore, it challenges theories of mechanotransduction that are based strongly on "known" discrepancies between *in vitro *and *in vivo *stress regimes, since presumed controlled stress regimes *in vitro *are likely to be different than those applied in these theories. Spatial variance in target stresses underscore this point. Based on the data of this study, there is significant spiatial variance in shear stresses from the target value and there may only be a small area within the chamber in which the desired stresses are imparted. Hence, the assumption that stresses found in the center of the chamber accurately reflect the target value may be invalid. However, flow field simulations provide insight into local stresses imparted at cell surfaces, providing a unique perspective for elucidating mechanotransduction at the cellular level. If one is aware that stresses vary spatially within and between chambers, flow simulation models could be exploited to identify relevant areas of interest for specific outcome measures.

This study was implemented using a target shear stress of 1 and 10 dyn/cm^2^, and the results found for the chambers were comparable to this value. However, the actual stress felt by osteoblasts and osteocytes *in vivo *remains unclear, and a large range of stresses has been applied in past studies. Furthermore, flow chambers are used in a wide variety of experiments with different kinds of cells. For example, endothelial cells have been subjected to shear stresses around 20 dyn/cm^2 ^[31,34,44] At this value, simulations for the chambers examined in this study showed a significant increase in the range of shear stresses experienced. Thus, it can be inferred that, as target stresses are increased, the effects on flow profiles and shear ranges would be amplified and problems inherent to identifying the location of the target stress location would increase as well. Therefore, the effects observed in these computational and experimental studies are expected to occur for a variety of target stresses.

Potential limitations in this study are derived from the fact that it is by nature a computational study which was validated experimentally using a steady flow condition. However, the simulations presented in this study are accurate, virtual depictions of the three commercial devices in use and allow for elucidation of flow regimes that would not be possible with current experimental fluid dynamics methods. A further limitation of the study was the idealization of the cell monolayer as an evenly spaced array of rigid, spheroid bodies, excluding detailed cellular structures. In addition, the cells were modeled as static entities, *i.e*. the model did not account for adaptation in cell structure or realignment of groups of cells with time, both of which have been observed experimentally in response to flow. Nonetheless, inclusion of the monolayer in the computational model resulted in prediction of shear stress magnitudes consistent with those reported in the literature for a similar geometry and setup [45].

With regard to the experimental studies, potential limitations were inherent to idealizations used in implementing the *μ*PIV protocol and within the flow chambers themselves. Due to the size of the particles relative to the cells, it was not feasible to use *μ*PIV to observe deviations in the flow attributable to the presence of the cell monolayer. Furthermore, when implementing all three commercial chambers, it was difficult to prevent completely leakage around the flow channels. Leakage reduces the effective flow rate through the channel (versus what is applied via the pump), and thereby reduces shear rates. In addition, for two of the chambers, the inlet and outlet areas were so small as to necessitate small caliber tubing that was prone to collapse and difficult to manipulate; this small diameter tubing was prone to very high fluid pressures, which sometimes caused the tubing to disengage from either the inlet/outlet or the syringe, resulting in persistent fluid leakage. Choice of gasket thickness could also influence both computational and experimental results as well and further complicate the targeting of particular flow or stress regimes. Finally, the formation of air bubbles appeared to be inherent to implementation of the types of parallel plate flow chambers studied in combination with a syringe pump; not only do such bubbles have the potential to disrupt the biochemical environment of the cells seeded in the chamber, but they also have the potential to disrupt the mechanical environment of the cells. In carrying out these studies, every effort was made to mitigate bubble formation and experiments were repeated if bubble formation was observed to disrupt flow.

## Conclusion

The results of this study challenge the basic premise of *in vitro *mechanotransduction studies, *i.e*. a controlled flow regime is applied to impart a defined mechanical stimulus to cells, even if it is not always possible to insure that the flow regimes are purely physiologic. In fact, flow regimes found in commercially available perfusion chambers are not constant and shear stresses that are imparted to cells are location dependent at the cellular level. Hence, cells on one side of a chamber may experience a different stress than those on the opposite side. This complicates the elucidation of cellular mechanisms of mechanotransduction. Furthermore, these flow fields differ between chambers as well; according to their geometry and set flow rate. This further exacerbates meaningful elucidation of mechanotransduction mechanisms through comparison of studies conducted with different chamber designs. At the very least, this study underscores the importance of calibrating devices to achieve stress magnitudes near targeted stress levels. From a broader perspective, by coupling computational fluid dynamics with cell biology, new approaches can be developed to overcome limitations of the current technology. Thereby, the impact of *in vitro *studies will be increased and data from different laboratories will be able to be compared, which could greatly increase the impact of cell biology research.

## Competing interests

Since completion of the study reported in this manuscript, the senior author has negotiated a licensing agreement with Warner Instruments to speed translation of intellectual property that will improve flow chamber performance. To date (9 February 2006), none of the authors has received reimbursements, fees, funding, or salary from Warner Instruments.

## Authors' contributions

EA carried out computational fluid dynamics studies and drafted the manuscript. TF helped to develop the experimental protocol, co-wrote automation and image analysis routines, carried out experimental PIV studies and helped draft the manuscript. AS helped develop PIV experimental protocols, co-wrote automation and image analysis routines, wrote the data management and visualization Mathematica notebooks, and helped draft the manuscript. MKT conceived of the study, coordinated its design, as well as drafted and revised the manuscript.
